# Impact of the COVID-19 Pandemic on Seasonal Variations in Childhood and Adolescent Growth: Experience of Pediatric Endocrine Clinics

**DOI:** 10.3390/children8050404

**Published:** 2021-05-17

**Authors:** Jin-Ah Han, Yae-Eun Chung, In-Hyuk Chung, Yong-Hee Hong, Sochung Chung

**Affiliations:** 1 Department of Pediatrics, Konkuk University Medical Center, Konkuk University School of Medicine, Seoul 05030, Korea; 20190107@kuh.ac.kr (J.-A.H.); 20160090@kuh.ac.kr (Y.-E.C.); 2 Department of Pediatrics, National Health Insurance Service Ilsan Hospital, Goyang 10444, Korea; ped-endo@nhimc.or.kr; 3 Department of Pediatrics, Soonchunhyang University Bucheon Hospital, Soonchunhyang University School of Medicine, Bucheon 14584, Korea; hongyonghee@schmc.ac.kr; 4 Research Institute of Medical Science, Konkuk University School of Medicine, Seoul 05029, Korea

**Keywords:** COVID-19, children, body mass index, seasonal variation, growth

## Abstract

Background: Children experience seasonal variations in growth whereby height increases most in spring and least in autumn, and weight increases least in spring and most in autumn. We hypothesized that activity restriction caused by efforts to contain the spread of coronavirus disease 2019 (COVID-19) would result in increased body mass index (BMI) in children, differing from conventional seasonal growth variations. Methods: We included 169 children who visited endocrine clinics of three hospitals in Korea at regular intervals under the same conditions for two years. Visit dates were D1 (January, 2019), D2 (July, 2019), D3 (January, 2020) before the COVID-19 outbreak, and D4 (July, 2020) during the pandemic. Differences in the z-score for height (HT), weight (WT), and BMI among time points and between spring seasons (i.e., S1–S3) were compared. Results: There were significant differences in BMIz among time points, which decreased from D1–D2 and increased from D2–D3 and D3–D4. WTz significantly increased from D2–D3 and D3–D4. BMIz values of S1 (spring 2019) and S3 (spring 2020) were −0.05 and 0.16, respectively, showing significant differences. WTz values between S1 and S3 were significantly different (−0.02 vs. 0.13). Conclusions: In 2019, there were conventional seasonal variations in BMIz, which declined in spring and increased in autumn, while in 2020, BMIz increased even in spring. The COVID-19 pandemic may have affected seasonal variations in the growth of children attending endocrine clinics.

## 1. Introduction

Coronavirus disease 2019 (COVID-19), caused by a new coronavirus called severe acute respiratory syndrome coronavirus 2 (SARS-CoV-2), is prevalent worldwide, and the World Health Organization (WHO) first learned of this new virus on 31 December 2019 and declared the novel coronavirus (COVID-19) outbreak a global pandemic on 11 March 2020 [1,2]. According to the WHO announcement on 6 December 2020, the cumulative number of confirmed cases worldwide is approximately 66 million, and the cumulative death toll is approximately 1.5 million [3]. As of 10 December 2020, more than 50 vaccines are in clinical trial stages worldwide, and Pfizer vaccination administration has already begun as of 8 December in the United Kingdom [4]. However, it will take a long time to reach population immunity by vaccination, and sufficient vaccine quantities have not yet been supplied to the world. Accordingly, COVID-19 is expected to continue to be prevalent in many countries for the foreseeable future.

In addition to vaccination, many countries, including Korea, are making efforts to prevent its spread. These measures include making it mandatory to wear a mask in indoor and outdoor public places, closing schools and replacing them with online classes, and restricting the use of stores and sports centers [5]. As a result of these measures, the activities of most people, including children, have been restricted [6,7].

Season and time of year are among the environmental factors that influence the growth and development of children and adolescents [8,9,10,11,12,13,14,15,16]. There are seasonal variations in growth, in that height increases most in spring and least in autumn, and weight increases least in spring and most in autumn [8,9,10,11]. In a recent cross-sectional study, there was a rapid decrease in BMI z-scores from May to June and a rapid increase between August and September [13]. Body mass index (BMI) in children is known to increase more during vacation periods than school semesters [17,18]. One of the reasons for this is decreased physical activity during the vacation [17]. Concerns about weight gain have increased after the COVID-19 outbreak, along with decreased physical activity and the restriction of outdoor activity, in the era of an obesity epidemic.

We conducted this study to confirm whether activity restriction caused by efforts to contain the spread of COVID-19 may cause weight gain and result in increased BMI in children and adolescents, and to determine whether there was a different seasonal variation after the COVID-19 outbreak in children who visited endocrine clinics.

## 2. Materials and Methods

### 2.1. Subjects

This study included 169 children and adolescents (40 males, 129 females) who visited pediatric endocrine clinics of three hospitals (Konkuk University Medical Center, National Health Insurance Service Ilsan Hospital, Soonchunhyang University Bucheon Hospital) in Korea at regular intervals between January 2019 and July 2020.

Most of the participants were children who visited the outpatient clinic for monitoring of growth and puberty. The reasons for visits were categorized into monitoring of growth (18.3%) or pubertal development (23.7%), the diagnosis of early or precocious puberty (41.4%), growth hormone deficiency (7.1%), or other endocrine disorders (9.5%) such as congenital hypothyroidism on levothyroxine with stable euthyroid state. In addition, patients receiving a gonadotropin-releasing hormone (GnRH) agonist for precocious puberty were included in the study.

Patients who had the same treatment for their individual disease for the study duration were included, and patients whose treatments changed were excluded. The study protocol was approved by the institutional review board of Konkuk University Medical Center (file No.: 2020-12-008).

### 2.2. Anthropometric Measurements

Height and weight were measured in light clothing without shoes. Height was measured with a stadiometer (SECA 225, SECA Deutschland, Hamburg, Germany) to the nearest 0.1 cm, and weight was measured with a digital scale (GL-6000-20, CASKOREA, Seoul, Korea) to the nearest 0.1 kg. BMI was calculated as weight in kilograms divided by height in meters squared. Height (HT), weight (WT), and BMI z-scores were calculated with the LMS method by age and sex, using the 2017 Korean National Growth Charts for children and adolescents [19].

**Figure 1:**
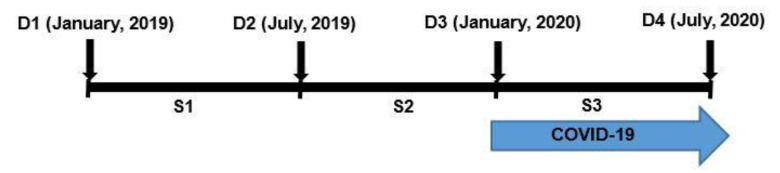
Schematic diagram of the dates of visits (D) and seasons (S).

The dates of visits (D) on which the anthropometric measurements were taken were D1 (January, 2019), D2 (July, 2019), D3 (January, 2020) before the COVID-19 outbreak, and D4 (July, 2020) after the peak of the outbreak, for a total of four visits. In addition, we also calculated the season (S), which is the change in growth value for each visit point. A schematic diagram of D (date of visit) and S (season) is shown in Figure 1. We defined the spring season as from January to July, and the fall season from July to January of the following year, with the study divided into three seasons (i.e., S1, S2, and S3).

Thereafter, differences in the z-score of height, weight, and BMI among the time points (i.e., D1–D2, D2–D3, and D3–D4) and between spring 2019 and spring 2020 (i.e., S1–S3) were compared and analyzed.

### 2.3. Statistical Analysis

Each result was expressed as the mean ± standard deviation. The z-scores for height, weight, BMI per visit date (D), and season (S) were compared and analyzed using a paired *t*-test. All analyses were performed using SPSS ver. 17.0 (SPSS Inc., Chicago, IL, USA), and *p*-values of less than 0.05 were considered significant.

## 3. Results

A total of 169 children were included for analysis. The demographic data of the study group by sex at the first visit (D1) are summarized in Table 1. The mean age was 11.14 ± 2.44 years and 9.56 ± 2.07 years for boys and girls, respectively, and the mean overall age was 9.93 ± 2.26 years. The mean BMI was 19.04 ± 2.84 kg/m^2^, and the mean BMIz was 0.41 ± 1.01 kg/m^2^ for all participants.

**Figure 2:**
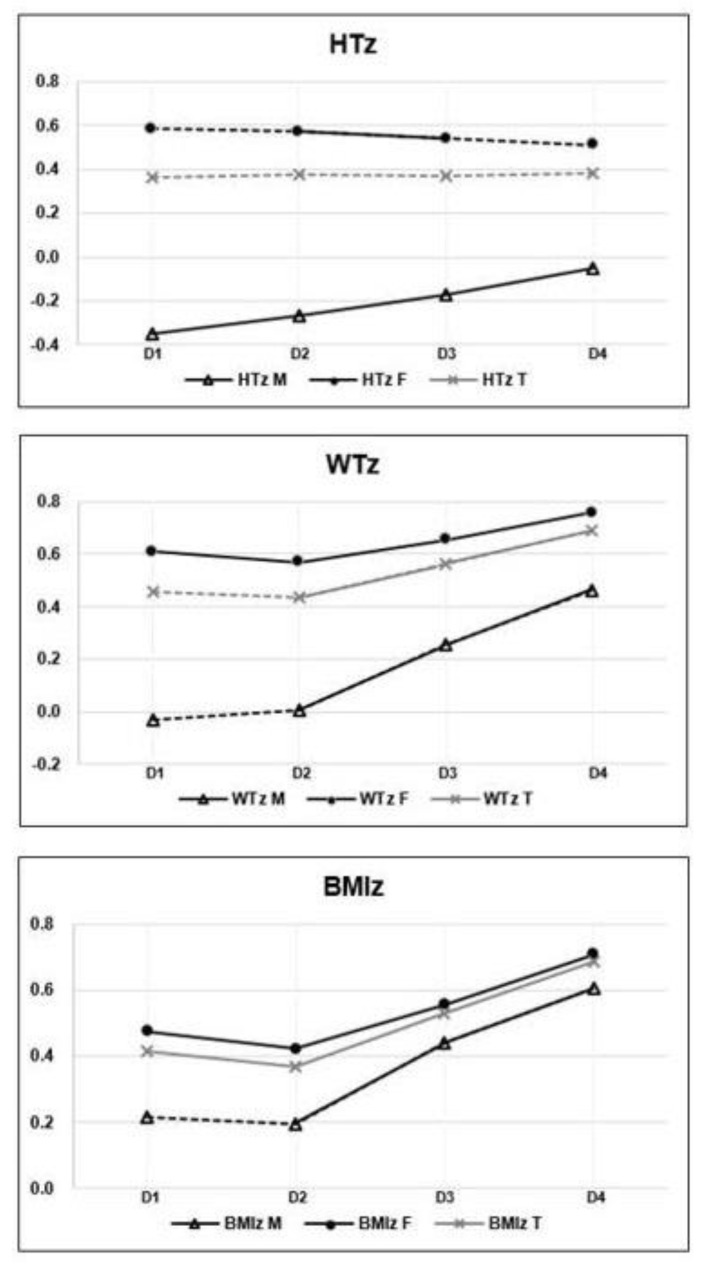
Z-scores of height (HT), weight (WT), and body mass index (BMI) at each visit date (D). D, date of visit; M, male; F, female; T, total; HTz, height z-score; WTz, weight z-score; BMIz, body mass index z-score. The solid line indicates that the difference in the value among the dates of visits is statistically significant, and the dotted line indicates non-significant differences.

The comparisons of z-scores for height, weight, and BMI among the visit dates are shown in Figure 2 and Table 2. The averages of HTz, WTz, and BMIz at each visit date (D) are shown in the form of a line graph in Figure 2. In this graph, the solid line indicates that the values among the time points were significantly different (*p* < 0.05), and the dotted line indicates that the difference was not significant (*p* > 0.05). HTz significantly increased across visit dates in boys, but there was no significant difference between any visit dates for the cohort overall. With regard to WTz and BMIz changes, they were not significantly different between D1 and D2 in boys, but increased significantly between D2 and D3 and between D3 and D4. In girls, WTz and BMIz significantly decreased between D1 and D2, and significantly increased between D2 and D3 and between D3 and D4. For the study cohort, the averages of BMIz in D1 and D2 were 0.41 ± 1.01 and 0.37 ± 1.04, and BMIz significantly decreased from D1 to D2 (*p* = 0.047), as shown in Figure 2 and Table 2. The means of BMIz in D2, D3, and D4 were 0.37 ± 1.04, 0.53 ± 1.03, and 0.68 ± 1.12, respectively, and there were significant increases observed from D2–D3 and D3–D4 (*p* < 0.001).

The comparisons of z-scores between the spring seasons (S1, spring 2019 vs. S3, spring 2020) are shown in Table 3. For the total study cohort, no significant difference was observed between S1 and S3 for HTz, but the means of WTz were significantly different between S1 and S3 (*p* < 0.001), at –0.02 ± 0.24 and 0.13 ± 0.28, respectively. The averages of BMIz in S1 and S3 were −0.05 ± 0.30 and 0.16 ± 0.34, respectively, for which the difference was statistically significant (*p* < 0.001).

## 4. Discussion

The worldwide impact of the COVID-19 pandemic has continued for more than a year, and negative health effects secondary to restrictions may be exacerbated. In this study, we hypothesized that activity restriction as a result of efforts to prevent the spread of COVID-19 would result in more weight gain and higher BMI, leading to a difference in growth from the conventional seasonal variations in children.

The strength of this study is that the data collected were reliably measured directly by medical institutions. We confirmed the existence of conventional seasonal variation in growth before the COVID-19 pandemic and different patterns after the COVID-19 outbreak by comparing and analyzing the data obtained before and after the COVID-19 outbreak in children attending endocrine clinics.

Although some studies have suggested and reported increased weight and BMI secondary to the COVID-19 pandemic, these studies were conducted in the form of questionnaires instead of measuring weight and height; thus, the studies may have been affected by bias and low reliability [7,20,21,22].

A previous study predicted the impact of the COVID-19 pandemic on childhood obesity using a microsimulation model [23]. The predicted increase in the BMIz in scenarios 1 to 4 were 0.056, 0.084, 0.141, and 0.198, respectively. Increased BMIz in S3 in our study was 0.16, which is comparable to scenarios 1 to 4. Considering that our study ended in July 2020, it can be seen that the impact of the COVID-19 pandemic is greater than expected, with a value greater than 0.141 in scenario 3.

As shown in Figure 2 and Table 2, in the total study cohort, there were significant differences in BMIz among the time points, decreasing in D1–D2 and increasing from both D2–D3 and D3–D4. This suggests that in 2019, there were seasonal variations in BMIz, which declined in spring and increased in autumn, as in previous studies [8,9,10,11]. Meanwhile, in 2020, after the peak of the COVID-19 outbreak, BMIz increased even in spring, contrary to the typical seasonal variation.

In addition, there were significant differences (*p* < 0.001) between S1 (2019 spring) and S3 (2020 spring) in WTz and BMIz in the overall cohort, as shown in Table 3. In S1, the means of WTz and BMIz were −0.02 and −0.05, respectively, compared to those of S3 at 0.13 and 0.16, respectively. That is, WTz and BMIz decreased in the spring season before the COVID-19 outbreak, but increased in the spring season after the peak of the outbreak. These results suggest that the COVID-19 pandemic affected the seasonal variations in this population.

This study had several limitations. First, this study was conducted under the assumption that physical activity would have decreased due to the COVID-19 outbreak. However, there is no analysis of physical activity before and after the COVID-19 outbreak; thus, the causal relationship may not be clear. Second, our study period was not long enough and only the first half of the year after the start of the COVID-19 pandemic was investigated.

Third, the sample may not be fully representative, as the sample size was small, and only children who visited the pediatric endocrine outpatient clinics, including for growth and puberty monitoring, were included. The incidence and prevalence of central precocious puberty (CPP) in Korea have increased steeply in recent years, especially in girls [24,25]. According to a study investigating the incidence and prevalence of CPP from 2008 to 2014, the incidence and prevalence of CPP was two to three times higher than those of a Danish study investigating the period from 1993 to 2001 [25,26].

Most (90.5%) of the subjects in this study visited an outpatient clinic for the evaluation of growth and puberty development, including those treated for growth hormone deficiency and CPP, and girls accounted for the majority (76.5%) of the population. Forty percent of boys visited for growth monitoring, while 77.5% of girls for pubertal development owing to a concern of early or precocious puberty. Since there were no changes in or discontinuations of treatment during the study period, including these patients did not have an effect on the results of this study.

The high interest in growth and puberty could be a socio-cultural characteristic among Koreans. Thirty years ago, type 2 diabetes in children was considered rare [27]. However, the obesity epidemic has led to a dramatic increase in the numbers of youth with obesity-related disorders all over the world, including Korea [28,29,30]. Rising rates of obesity-related disorders in Korean children have attracted the attention and concerns of parents and society. The rate of dyslipidemia in obese Korean adolescents is high and has been reported as 56.1%, and the 2017 clinical practice guidelines for dyslipidemia of Korean children and adolescents have been published [29,31]. Limited studies are available to define the risks of obesity-related comorbidities in Korean children and more studies are needed [32,33].

Weight gain after the COVID-19 pandemic may be due to a decrease in physical activity, and other factors, such as increased sedentary behavior or increased access to unhealthy foods, may also have contributed. It is already known that children’s weight and BMI increase more during vacation periods compared to during school semesters [17,18]. One of the reasons for weight gain during vacations is a decrease in physical activity [17]. Therefore, through this study, it is possible that COVID-19-related activity restriction may cause weight gain in children and adolescents attending endocrine clinics that differs from typical seasonal variations. Nonetheless, for a clearer causal relationship, a quantitative analysis of physical activity before and after COVID-19 is needed.

## 5. Conclusions

In 2019, there were seasonal variations in BMIz, which declined in spring and increased in autumn, while, in 2020 during the COVID-19 pandemic, BMIz increased even in spring. Our data suggest that the COVID-19 pandemic may have affected the seasonal variations in growth in this population. Further investigation is required to elucidate the long-term effects of the COVID-19 pandemic on growth in children.

## Figures and Tables

**Table 1:** The characteristics of the study populations at the first visit (D1).

	Male (n = 40)	Female (n = 129)	Total (n = 169)
Age (yr)	11.1 ± 2.4	9.6 ± 2.1	9.9 ± 2.3.
HT (cm)	144.2 ± 17.4	138.3 ± 9.9	139.7 ± 12.3
WT (kg)	43.2 ± 16.1	36.4 ± 8.8	38.0 ± 11.3
BMI (kg/m^2^)	20.0 ± 3.6	18.8 ± 2.5	19.0 ± 2.8
HTz	−0.35 ± 1.25	0.58 ± 1.14	0.36 ± 1.23
WTz	−0.03 ± 1.32	0.61 ± 0.97	0.46 ± 1.09
BMIz	0.22 ± 1.17	0.47 ± 0.95	0.41 ± 1.01

Values are presented as means ± standard deviations. HT, height; WT, weight; BMI, body mass index; HTz, height z-score; WTz, weight z-score; BMIz, body mass index z-score.

**Table 2:** The comparisons of z-scores among the visit dates (D).

		D1	D2	D3	D4
HTz	M ^abc^	−0.35 ± 1.25	−0.27 ± 1.24	−0.17 ± 1.12	−0.05 ± 1.09
	F ^b^	0.58 ± 1.14	0.57 ± 1.10	0.54 ± 1.08	0.51 ± 1.06
	T	0.36 ± 1.23	0.37 ± 1.18	0.37 ± 1.13	0.38 ± 1.09
WTz	M ^bc^	−0.03 ± 1.32	0.01 ± 1.31	0.26 ± 1.29	0.46 ± 1.40
	F ^abc^	0.61 ± 0.97	0.57 ± 1.00	0.65 ± 0.99	0.76 ± 1.02
	T ^bc^	0.46 ± 1.09	0.44 ± 1.10	0.56 ± 1.08	0.69 ± 1.12
BMIz	M ^bc^	0.22 ± 1.17	0.19 ± 1.16	0.44 ± 1.21	0.61 ± 1.36
	F ^abc^	0.47 ± 0.95	0.42 ± 1.00	0.56 ± 0.98	0.71 ± 1.04
	T ^abc^	0.41 ± 1.01	0.37 ± 1.04	0.53 ± 1.03	0.68 ± 1.12

Values are presented as means ± standard deviations. D, date of visit; M, male; F, female; T, total; HTz, height z-score; WTz, weight z-score; BMIz, body mass index z-score. The *p*-value of the difference among the visit dates was calculated using a paired *t*-test. ^a^: *p*-value was statistically significant (<0.05) between D1 and D2, ^b^: *p*-value was statistically significant (<0.05) between D2 and D3, ^c^: *p*-value was statistically significant (<0.05) between D3 and D4.

**Table 3:** The comparisons of z-scores by spring season (S1 (2019 spring) vs. S3 (2020 spring)) using paired *t*-test.

		S1	S3	*p* Value
HTz	Male	0.08 ± 0.19	0.12 ± 0.23	0.236
	Female	−0.01 ± 0.17	−0.03 ± 0.18	0.350
	Total	0.01 ± 0.18	0.01 ± 0.20	0.882
WTz	Male	0.04 ± 0.27	0.20 ± 0.35	0.025
	Female	−0.04 ± 0.23	0.10 ± 0.25	<0.001
	Total	−0.02 ± 0.24	0.13 ± 0.28	<0.001
BMIz	Male	−0.02 ± 0.31	0.17 ± 0.42	0.027
	Female	−0.05 ± 0.29	0.15 ±0.32	<0.001
	Total	−0.05 ± 0.30	0.16 ± 0.34	<0.001

Values are presented as means ± standard deviations. HTz, height z-score; WTz, weight z-score; BMIz, body mass index z-score; S, season; S(n) z-score = (D(n + 1) z-score) – (D(n) z-score). The *p*-value of the difference between S1 and S3 was calculated using a paired *t*-test.

## Data Availability

The data presented in this study are available on request from the corresponding author. The data are not publicly available due to personal medical data.
